# Characterization of *Boswellia rivae* Engl Resin as a Potential Use for Pharmaceutical Excipient

**DOI:** 10.1155/2022/5791308

**Published:** 2022-08-08

**Authors:** Fanta Gashe, Desta Assefa, Shibiru Tesema, Gemechu Zeleke, Ramanjireddy Tatiparthi, Dereje Kebebe, Sultan Suleman

**Affiliations:** ^1^School of Pharmacy, Institute of Health, Jimma University, Jimma, Oromia, Ethiopia; ^2^Jimma University Laboratory of Drug Quality (JULaDQ), Jimma, Oromia, Ethiopia

## Abstract

Pharmaceutical excipients derived from natural sources like resins are nowadays meritoriously used in the formulation of drugs. Resins of natural origin have many advantages over chemically synthesized substances; they are safer, nontoxic, less expensive, biodegradable, and widely available. To our knowledge, resins from plants have been not sufficiently explored for application in pharmaceutical formulations. Thus, in the present study, a resin isolated from *Boswellia rivae* Engl was characterized for its potential use as a pharmaceutical excipient. *Method*. The resin was extracted from the oleo gum resin of *Boswellia rivae* Engl, which involved the removal of volatile oils, gum, and Boswellic acid contents. The dried resin powder was then characterized for its micromeritic properties, heavy metal contents, moisture content, moisture absorption power, pH, solubility, swelling property, and acute toxicity profile. Moreover, the crystal nature and the chemical functionality of the resin were evaluated by using X-ray diffraction and Fourier transform infrared spectrometry, respectively. *Results*. The yield of the neutral resin was 13.17%, and the powder was pale yellow and had irregular surfaces. The resin was freely soluble in organic solvents but almost insoluble in water. The moisture content of the dried extract was 2.5% while its moisture absorption capacity was 2.5%, 4%, and 5.47% at 40%, 60%, and 75% RH, respectively. Besides, the maximum swelling capacities of the resin observed were 40%, 37%, and 30% at 350C, 300C, and 250C, respectively. The bulk powder exhibited a 1.21 Hausner ratio, 36.497 angles of repose, and 17.03% Carr's index, indicating the fair flowability of the powder. Heavy metals such as zinc, chromium, and cobalt were detected at a low level while elements like copper, manganese, lead, and cadmium were absent. The X-ray diffraction study revealed that the crystallinity index of the powder was 42.7% with a crystal size of 994.5A. The *Boswellia* resin could be safe in mice up to 3 g/kg of their body weight. In conclusion, the physicochemical properties of the resin powder investigated reveal its potential application as pharmaceutical additives in the formulation of modified release solid dosages forms like tablets and microcapsules.

## 1. Introduction

Natural resins are complex substances that are produced as oxidation products of essential oils and act in response to ecological interactions. Resins are applied in different areas including in the pharmaceutical sector as excipients in the formulation of various dosage forms [1, 2]. The functions of pharmaceutical excipients including resins are to control the release of drugs, maintain stability, alter the bioavailability, improve the therapeutic efficacy of drugs, and decrease unwanted side effects [3, 4]. Unfortunately, there are very few natural resins that have been explored for use as additives in pharmaceuticals. For instance, rosin is among the very few resins that are applied for pharmaceutical applications [5, 6]. Thus, natural products are still the potential sources for the further investigation of resins that can be applied to the pharmaceutical sector.

Natural resins can be directly obtained either from plants or animals [7]. They are, however, mostly extracted and isolated from plants [8]. Like other natural additives, natural resins have many advantages over chemically synthesized substances; they are safer, less expensive, biodegradable, and widely available [9, 10]. Natural resins could be thus a good choice to be used as excipients in various pharmaceutical dosage forms, like in the preparation of microcapsules, as film formers, coating materials, and matrix formers in solid dosage forms, as well as in drug delivery systems [11, 12].

The *olibanum* or oleo gum resin which is obtained from *Boswellia* species consists of three components; resins, volatile oil, and gum. The resin portion composes of mainly pentacyclic triterpenes and exists in large proportions (30-60%) compared to the other ingredients [13]. The harvesting method of the resins does not harm the plants' sources since it can be obtained by scraping off the plant with iron or collected simply on the mats when it falls on the ground [14]. This makes it suitable for bulk production from locally available sources once its pharmaceutical application is approved.

There had been limited studies conducted before on the use of natural resins in general and *Boswellia* resins in particular as a pharmaceutical ingredient. A few studies revealed the potential use of the crude *Boswellia* gum resins as binding agents and drug release retardants [15, 16]. Nevertheless, the crude resin extracts of *Boswellia* species contain the potent bioactive compounds and Boswellic acids [17, 18]. Therefore, it is not appropriate to be used as an additive in the formulation of drug products owing to the fact that pharmaceutical excipients should be pharmacologically inert at used amounts. Considering this limitation, the isolated resin from the oleo gum resin of *Boswellia rivae* Engl was investigated after separating the bioactive Boswellic acid contents using a standard method. Thus, the physicochemical properties of the neutral resin were evaluated for its potential use as an excipient in the formulation of drugs.

## 2. Methods

### 2.1. Instruments and Materials

Atomic absorption spectrometer (novAA 400p, 07745 Jena, Germany), analytical balance (Mettler Toledo, Columbus, OH 43240, USA), sieving mesh (Fritsch, Idar Oberstein, Germany), drying oven (Memmert, Germany), Whatman no. 4 filter paper (Whatman International Ltd, England), pH meter (Metkorp Equipment Pvt. Ltd, Hungary), X-ray diffractometer (Drawell XRD 7000, Shanghai, China), and Fourier transform infra-red spectroscopy (PerkinElmer, Spectrum Two, Waltham, MA 02451 USA) were used.

### 2.2. Chemicals

Ethyl acetate, ethanol, chloroform, acetone, hydrochloric acid, ferric chloride, copper sulfate, sulfuric acid, and sodium potassium tartrate were from LOBA Chemie, India; potassium hydroxide, sodium hydroxide, sulfuric acid, hydrochloric acid, and nitric acid were from CDH LTD, India; hydrogen peroxide was from Alpha Chemica, India; iodine, potassium bromide, and potassium iodide were from Merck, Germany; and distilled water was from JULaDQ, Ethiopia. All the reagents and solvents were analytical grade.

### 2.3. Extraction and Isolation of the Resin

The *Boswellia rivae* Engl plant material was obtained from Ethiopian Natural Gum Processing and Marketing Enterprise (NGPME). The plant authentication was performed at Addis Ababa University by the Department of Biology. The powdered oleo gum resin (50 g) was subjected to hydrodistillation after dispersing in 150 mL water using a Clevenger's apparatus to remove the volatile components. After cooling to room temperature (25°C), the remaining residue containing the water-resin mixture was extracted twice with 100 mL of ethyl acetate. The crude resin was then obtained after drying the organic layer by evaporation of the solvent in a vacuum. This was followed by constantly washing the residue with 100 mL of hot water multiple times to remove the traces of the gum with consequent drying at room temperature. The dried crude resin was subsequently treated with 0.1 M KOH to remove the acidic fraction, Boswellic acid. This resulted in the separation of the precipitated neutral resin from the water-soluble fraction containing Boswellic acid. This process was done in triplicate. The precipitated resin was separated and further washed with 50 mL of distilled water exhaustively to remove the alkali and the other water-soluble impurities. Finally, the neutral resin was dried at room temperature and kept in the air-tight amber glass containers in the fridge at 8°C for a maximum of a year until further investigations [19, 20].

### 2.4. Phytochemical Screening

The resin powder from *Boswellia rivae* Engl was evaluated qualitatively for the presence of plant metabolites. The standard procedures were applied to determine the alkaloid, carbohydrate, protein, phenol, glycoside, flavonoid, saponin, and tannin [21, 22].

#### 2.4.1. Test for Phenol

After dissolving about 50 mg of the powder in 5 ml of distilled water, a few drops of 5% ferric chloride solution were added. The development of a dark green color was regarded as a positive test for phenolic compounds.

#### 2.4.2. Test for Glycosides

To 20 mg of the resin powder, 2 ml glacial acid and and one drop of ferric chloride were added. Consequently, it was observed for the development of a brown ring at the interface after the addition of 1 ml of concentrated sulfuric acid as it could reveal the presence of glycosides.

#### 2.4.3. Test for Proteins

About 1 ml of 4% NaOH solution and a few drops of 1% CuSO_4_ were mixed with the test solution (2 m). Then, it was observed for the development of violet color which could show the presence of protein.

#### 2.4.4. Test for Alkaloids

The dispersion of resin powder in 2% H_2_SO_4_ was warmed for two minutes. After filtration, a few drops of the Wagner's reagent were added, and the development of a reddish-brown precipitate was regarded as a positive test for alkaloids.

#### 2.4.5. Test for Tannins

About 100 mg of the resin was boiled in 2 mL of water/dimethyl sulfoxide with subsequent filtering. Then, a few drops of 0.1% of ferric chloride were added to the filtrate and observed for the brownish-green or a blue-black color formation.

#### 2.4.6. Test for Carbohydrates

The resin was dispersed in Molisch's reagent (*α*-naphthol dissolved in ethanol). Then, the development of a purple ring at the interface between the resin and the acid followed by the addition of a few drops of concentrated sulfuric acid was regarded as a positive test for carbohydrates.

#### 2.4.7. Test for Saponins

About 100 mg of the resin was dispersed in distilled water with subsequent boiling. The formation of foam on vigorous shaking after the addition of 3 drops of olive oil was considered to be an indicator of the presence of saponins.

#### 2.4.8. Test for Flavonoids (Alkaline Reagent Test)

A few drops of sodium hydroxide solution were added to the test solution. The formation of intense yellow color which turns colorless after the addition of a few drops of dilute acid was taken as a positive test for flavonoids.

### 2.5. Metallic Ion Content Analysis

The metallic contents of *Boswellia rivae* Engl resin were quantified using atomic absorption spectrometer (novAA 400p, 07745 Jena, Germany). About 6 ml of 2 M concentrated nitric acid and 2 ml of 30% H_2_O_2_ were added to 1 g of resin in 250 ml beakers and covered with a watch glass to digest the sample with great care on a hot plate in a fumed chamber. Then, the solutions were cooled slightly, and 30 ml of distilled water was added to each and boiled for about 10 minutes and filtered into a 100 ml volumetric flask using a Whatman no. 4 filter paper. Lastly, the solutions were made to the mark with distilled water before analysis [23].

### 2.6. Organoleptic Evaluation

The isolated resin was characterized for its organoleptic properties such as color, odor, and texture.

### 2.7. The Moisture Content of the Resin

The sample powder (2 g) was put into petri dishes and then heated in an oven at 105°C to a constant weight. The percentage loss of the moisture was then calculated using the following formula [16].

(1) $$\begin{array}{c} \text{Moisture content }\left(\%\right)=\frac{\text{Initial weight of the sample}-\text{weight after drying }}{\text{Initial weight of the sample powder}}*100. \end{array}$$

### 2.8. Relative Solubility of the Resin

The relative solubility of the resin powder was determined using a method described by Carter in various solvents such as in acetone, chloroform, ethanol, 0.1 M HCl, 0.1 M NaOH, and water. Accordingly, 1 g of resin was added to 10 ml of the abovementioned solvents and shaken for about 1 hr. Then, the mixtures were left overnight at a temperature of 25°C. Finally, a 5 ml clear supernatant was taken into small preweighed evaporating dishes and heated to dryness over a water bath for organic solvents and in an oven for aqueous solutions [24].

### 2.9. Swelling Capacity

The swelling index of the resin powder at various temperatures was determined by transferring 1 g of the resin powder separately into a 25 ml glass-stoppered graduated measuring cylinder, and the volumes occupied were noted. Subsequently, 20 ml of the solvents was added, and the cylinders were closed. These were shaken vigorously for 1 hour and then allowed to stand for 6 hours at various temperatures [25].

(2) $$\begin{array}{c} \text{Swelling index}=\frac{\text{Initial volume of the sample }}{\text{Final volume of the sample }}. \end{array}$$

### 2.10. The Moisture Absorption Capacity of the Resin

About 2 g of predried resin powder in the oven at 120°C was evenly distributed over the surface of Petri dishes and placed in a large desiccator at different relative humidity (RH = 40, 60, and 75%). The desiccator was stored at room temperature for seven days. The weight gained by the exposed sample was then recorded [26].

(3) $$\begin{array}{c} \text{Moisture sorption capacity }\left(\%\right)=\frac{\text{Weight after test}-\text{weight before test}}{\text{Weight of the sample before test}}*100. \end{array}$$

### 2.11. Angle of Repose

The static angle of repose “*θ*” was determined using the fixed funnel and free-standing cone method [23].

(4) $$\begin{array}{c} \text{Angle of Repose}\left(\tan\;\theta \right)=\frac{\text{Height of the cone}\left(H\right)}{\text{Radius of the cone}\left(R\right)}. \end{array}$$

### 2.12. Bulk and Tap Density

Bulk density was determined by pouring the powder into a graduated cylinder via a large funnel, and then, the volume and weight of the powder were recorded. Tapped density was determined by transferring a known mass of powder into a graduated cylinder, which was followed by mechanical tapping for a fixed number of taps until the powder bed volume reached a minimum level.

The Hausner ratio and Carr's index were calculated as follows [27].

(5) $$\begin{array}{c} \text{ Hausner ratio}=\frac{\text{ Tapped density}}{\text{Bulk density}},\\ \text{Car}{\text{r}}^{\prime }\text{s index}=\text{Tapped density}-\frac{\text{Bulk density}}{\text{Tapped density}}*100. \end{array}$$

### 2.13. Particle Size and Size Distribution Study

The particle size distribution of the resin was determined by sieve analysis using standard perforated plate sieves with mesh width from 5 *μ*-125 mm. The analysis was carried out by arranging the sieves in descending order of their aperture sizes [28].

### 2.14. Determination of the Functional Moiety

A small amount of the resin was blended with KBr (1 : 10) and compressed into discs on an IR press to serve as controls. The resultant discs will then be scanned on a PerkinElmer spectrum of two FT-IR spectrophotometers (Drawell XRD 7000, Shanghai, China) within a wavelength range of 400 cm^−1^ to 4000 cm [29, 30].

### 2.15. Determination of Solid-State Property

The powder sample was applied onto the sample holder and then subjected to X-ray diffractometer at a scanning rate of 0.02° using a Drawell XRD 7000 (PerkinElmer, Spectrum Two, Waltham, MA 02451 USA). Cu-Ka (3 kw) was used as a scanning source with a scanning range of 5°-80° at a scanning rate of 0.02/min. The crystalline index and crystal size were evaluated using Match software [31].

### 2.16. Acute Oral Toxicity Test

Acute toxicity test of the *Boswellia rivae* Engl resin was conducted in Swiss albino female mice having a weight range of 25 to 30 g. The resin powder was dissolved in a 1% Dimethyl sulfoxide (DMSO) solution. All the mice were placed in the appropriate animal cages under standard conditions, and they were allowed to have a standard diet and free water access for five days before analysis. On day six after abstaining from food but not water for 3 h, they were randomly divided into 2 groups (*n* = 6). Instantly, the first group (control group) received (0.25-0.3 ml) the vehicle per oral route (p.o.) while group 2 received similar volumes of a single dose of 3 g/kg resin via the same route. Then, the animals were observed for the appearance of any change in general behavior, weight, food consumption, physical activities, and mortality for continuous 14 days [26].

### 2.17. Statistical Analysis

The data were analyzed using Microsoft Excel, Origin Lab 2019b software (Origin Lab Corporation), and Match software version 3.12. The tests were conducted in triplicate, and the results were presented as mean and standard deviation.

## 3. Results

### 3.1. The Yield of the Resin

The yield of the *Boswellia rivae* Engl resin was calculated to be 13.17 g per 100 g of the oleo gum resin powder. The powder was pale yellow and had smooth surfaces.

### 3.2. Physicochemical Properties of the Powder

#### 3.2.1. Phytochemical Analysis

The qualitative evaluation of the phytochemicals revealed that there were trace amounts of carbohydrates, but most of the tests were negative for the majority of secondary metabolites (Table 1).

#### 3.2.2. Heavy Metal Content Analysis

Heavy metal analysis of the *Boswellia rivae* Engl resin extract was carried out to determine the concentration level of the elements. The standard calibration curves with R-square greater than 0.995 were applied to determine the concentration of the metal impurities in the samples (Figure S1). The experimental findings revealed the presence of zinc, chromium, and cobalt while the rest of the tested heavy metals were undetected at the tested concentration (Table 2).

#### 3.2.3. Relative Solubility, pH, and Loss on Drying

The pH of the aqueous dispersion of the resin powder was 7.1. Besides, its moisture content was found to be 2.5% (Table 3). The resin was freely soluble in organic solvents such as chloroform, acetone, and ethanol, but it was very slightly soluble in water and acidified water (Table 4).

#### 3.2.4. Moisture Absorption and Swelling Properties

**Figure 1:**
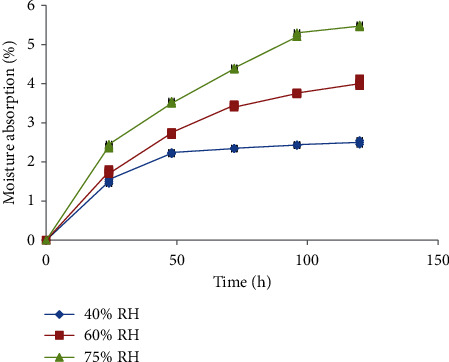
Moisture absorption properties of the resin at 25°C at various relative humidity (*n* = 3, mean ± SD).

**Figure 2:**
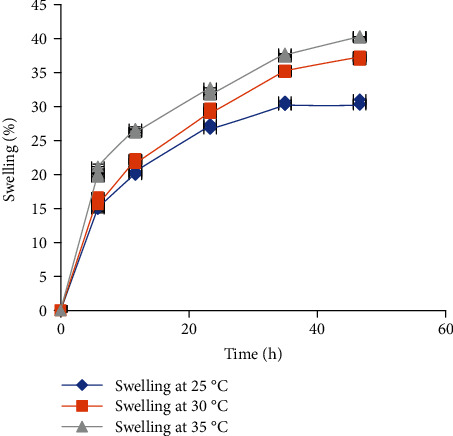
Swelling index of the Boswellia neutral resin at different temperatures (*n* = 3, mean ± SD).

The moisture absorption power of the *Boswellia rivae* Engl resin powder was evaluated at various relative humidity levels, and the results revealed that it absorbed the moisture steeply for the first 60 minutes, then decreased to a maximum level of 2.5%, 4%, and 5.47% moisture content at 40%, 60%, and 75% RH, respectively (Figure 1). Besides, the resin powder exhibited different swelling powers at various temperatures, with more swelling capability at high temperatures. Its maximum swelling capacity was determined to be 40%, 37%, and 30% at 35°C, 30°C, and 25°C of the storage temperature, respectively (Figure 2).

#### 3.2.5. Micromeritic Properties of the Resin

**Figure 3:**
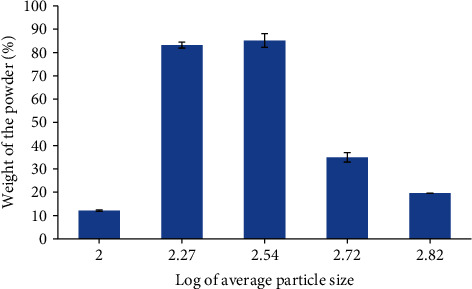
Particle size distribution of the Boswellia resin powder (mean ± SD).

The particle size distribution of the resin powder was within the size range of 75 *μ*m and 600 *μ*m. However, the particle size of the large proportion of the powder (71.67%) was between 125 *μ*m and 425 *μ*m. About 36.24% and 35.43% of the powder mass had an average particle size of 250 *μ*m and 125 *μ*m, respectively (Table 5). Moreover, the log plot of the particle size distribution revealed a unimodal distribution (Figure 3). The bulk densities of the whole powder and the fractions within the size range of 450-600 *μ*m were determined to be the same (0.57 g/ml). On the other hand, the tab density of the bulk powder (0.67 g/ml) was low in comparison to the sieve fractions of different size ranges. Similarly, the bulk powder exhibited a 1.21 Hausner ratio, a 36.497 angle of repose, and a 17.03% Carr's index, which were low in comparison to the other fractions with different size range obtained by sieving (Table 6).

### 3.3. Functional Moiety Determination and Solid State Properties

**Figure 4:**
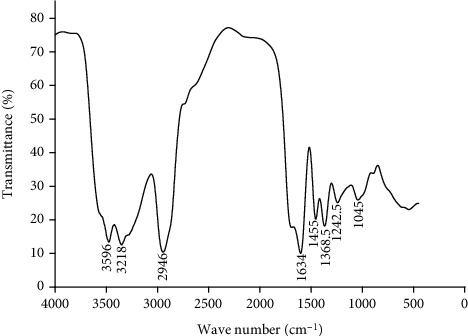
FTIR analysis of the resin powder.

**Figure 5:**
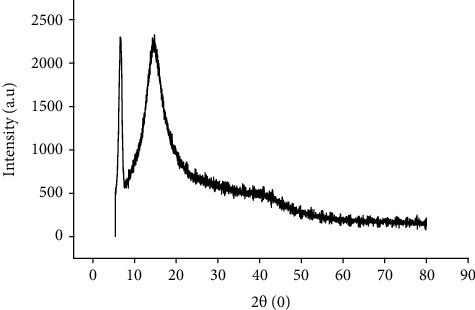
X-ray diffraction analysis of the resin.

FTIR spectrum revealed various peaks at 3596, 3218, 2946, 1634, 1455, 1368.5, 1242.5, and 1045 cm^−1^. The sharp peak at 3595 cm^−1^ and the broadband peak at 3218 cm^−1^ could be due to the stretching of O-H in alcohols and hydrogen-bonded OH stretching motion. The signals observed at 2946 cm^−1^ and 1633 cm^−1^ might represent the stretching vibration of C-H and C=C within the cyclic ring of the molecules. Small vibrations that could be corresponding to C-H bending, OH bending, and C-N stretching were also detected at 1455, 1368.5, and 1243 cm^−1^, respectively (27) (Figure 4). The X-ray diffraction analysis of the powder demonstrated peaks corresponding to Bragg's diffraction signals from the crystal plate. The peaks were observed at 11^o^ and 14^o^ angles with a total of 57.3% amorphous content. The crystal size of the particles was determined to be 994.5 A^0^ (Figure 5).

### 3.4. Acute Oral Toxicity Study

In acute oral toxicity study, there were no behavioral changes manifested by animals for the first 4 hours. The animals did not also show any sign of toxicity, and no mortality was recorded during the study period. Hence, there could be a possibility to use the resin as an additive in the formulations of drugs.

## 4. Discussion

Pharmaceutical excipients like resins can be obtained through chemical synthesis, directly from natural sources, or by chemical modification of natural products [32]. In this study, neutral resin was isolated from oleo gum resin, which was extracted from a plant called *Boswellia rivae.* The extract was a resinous, pale yellow irregular powder in appearance. The yield was found to be relatively fair, and thus reasonable for use as a pharmaceutical excipient. Moreover, the resin powder was devoid of the bioactive secondary metabolites, revealing its pharmacological inertness which is the main desired property of excipients used in drug formulations [33].

The moisture content of the pharmaceutical powder determines the compressibility of the powder in the granule and tablet formulation [34]. The presence of excess moisture in the powder causes cohesion by increasing the capillary forces and forming bridges among the particles. This affects the flowability and stability of the products [35]. The moisture content of the resin powder determined by loss on drying method was about 2.5% at ambient conditions, which is within the acceptance limit. However, this value could change depending on the relative humidity of the environment. As observed in the present study, the moisture-holding capacity of the powder increased as the humidity of the surrounding environment rose. Yet, its moisture content at high humidity was not beyond the desired limit to deter the quality of the product.

The solubility of pharmaceutical excipients can affect the delivery of the drugs from the dosage forms either by increasing or decreasing the release of the drugs from the dosage device [36]. The resin powder was found to be very slightly soluble in water even if it was freely soluble in various organic solvents. Less water-soluble polymers are usually employed in the formulation of medicine to sustain the release of drugs [37]. Besides, the powder exhibited swelling to some extent in water at a neutral pH value which allows slow diffusion of the candidate drugs after dissolution. On these grounds, *Boswellia* resin could be a potential excipient to be applied in the formulation of various modified-release products acting as a matrix former and microencapsulating agents.

The micromeritic properties of excipients can have an influence not only on the compounding procedure but also on the uniformity and quality of the final product [38]. The results of the micrometric properties study such as the angle of repose, the Hausner ratio, and the Carr's index of the bulk powder showed fair flowability. Consequently, the *Boswellia* resin powder without further processing or the addition of any flow promoters might be used as a pharmaceutical additive. The sieve analysis also revealed a narrow unimodal distribution of the powder. Thus, uniformity and the overall good processability of the product could be achieved during formulation.

Heavy metal analysis of the resin powder was carried out owing to the impact of these impurities on the safety of the patients. The heavy metals found in the resin powder were zinc, chromium, and cobalt, but very small amounts of these metals were detected, which does not raise any safety concerns. Furthermore, zinc and cobalt are class three metals; as a result, they are both low-hazard metals [39]. The acute toxicity study in mice also showed the absence of signs or changes in the behavior of the animals over the test period. All of these evidence could demonstrate the safety of the resin. However, it should be supported by organ and hematological tests.

X-ray diffraction analysis was applied to solid-state powder characterization to identify the crystal nature and polymorphism of the resin powder. The study results revealed that the powder was more amorphous with some segments of crystal nature. This is in agreement with the solid-state characteristics of most resins [11]. The FTIR analysis of the neutral resin revealed the presence of various functional groups. Similar findings were also reported in previous studies performed on crude extracts of *Boswellia* resin and isolated Boswellic acids [40, 41]. This shows the natural resin has a similar skeletal chemical structure to Boswellic acid; hence, it could be chemically triterpenoids. However, there were additional OH functional groups not observed in the previous studies, showing that one of the main components of the resin might be triterpenoid alcohols.

## 5. Conclusion

The isolated resin powder has been shown to exhibit fair flowability. The moisture content of the powder was within the acceptable range with some moisture absorption capacity that could assist compaction without having a significant effect on the flow and stability of the product. It was almost insoluble in water, but highly soluble in organic solvents, indicating that it could be applied in the formulation of sustained-release tablet dosage forms. A few heavy metals were detected in the powder in a very small concentration that does not raise any safety concerns. The study also revealed that the resin powder was amorphous and did not trigger any signs and symptoms in mice at 3 g/kg of their body weight. Overall, the observed characteristics of the resin reveal the potential application of the resin as a pharmaceutical excipient. However, further investigation is recommended to evaluate the organ toxicity profile of the resin in animals. The use of biodegradable, accessible, and economically cheap natural products in pharmaceuticals and other sectors is currently highlighted. Therefore, the major development in the future should focus on the evaluation of the usefulness of the resin as pharmaceutical additives by formulating it with model drugs after removing its bioactive components.

## Figures and Tables

**Table 1:** Phytochemical screening of the resin powder.

Phytochemicals	Tests	Test results
Phenols	Ferric chloride test	**—**
Glycosides	Sulfuric acid test	**—**
Proteins	Biuret test	**—**
Alkaloids	Wagner's test	**—**
Tannin	Ferric chloride test	**—**
Carbohydrates	Molisch's test	+
Flavonoids	Alkaline reagent test	**—**
Saponin	Foam test	**—**

- = absent; + = present.

**Table 2:** Heavy metal concentration of the resin.

Heavy metals	Standard conc. range (mg/l)	*R* ^2^ of the curve	Conc. of the sample (ppm)
Cd	0.02-3	0.9982	0.00
Zn	0.02-3	0.9997	0.14
Cr	0.02-3	0.9982	0.12
Pb	0.05-3	0.9960	0.00
Cu	0.02-3	0.9994	0.00
Co	0.02-3	0.9979	0.07
Mn	0.02-3	0.9950	0.00

**Table 3:** Physical properties of the resin powder.

Variables	Characteristics
Color	Pale yellow
Odor	Resinous
Texture	Irregular
Moisture lose on drying	2.5 ± 0.25%
pH	7.1 ± 0.015

*n* = 3, mean ± SD.

**Table 4:** Solubility of the Boswellia resin in various solvents.

Solvents	Solubility (g/ml)
Water	0.001 ± 0.000
Ethanol	0.083 ± 0.006
Acetone	0.093 ± 0.008
Chloroform	0.099 ± 0.002
0.1 M HCl	0.002 ± 0.000
0.1 M NaOH	0.006 ± 0.002

*n* = 3, mean ± SD.

**Table 5:** Particle size distribution of the Boswellia resin powder (*n* = 3, mean ± SD).

Screen opening (*μ*m)	Average mean of opening (*μ*m)	Weight of material retained (g)	Percentage weight	Cumulative % over weight	Cumulative % under weight
600	655	19.55 ± 0.03	8.32 ± 01	8.32	100
425	525	34.94 ± 0.16	14.87 ± 0.07	23.19	91.68
250	337.5	85.15 ± 0.18	36.24 ± 0.08	59.43	76.81
125	187.5	83.24 ± 0.28	35.43 ± 0.12	94.86	40.57
75	100	12.07 ± 0.35	5.14 ± 0.15	100	5.14

**Table 6:** Micromeritic property of the resin powder.

Variables	Particle size range (*n* = 3, mean ± SD)			
	Bulk powder	450-600 *μ*m	250-450 *μ*m	125-250 *μ*m
Bulk density (g/ml)	0.57 ± 0.01	0.57 ± 0.01	0.59 ± 0.01	0.48 ± 0.01
Tap density (g/ml)	0.67 ± 0.01	0.75 ± 0.01	0.75 ± 0.01	0.69 ± 0.01
Hausner ratio	1.21 ± 0.02	1.3 ± 0.03	1.27 ± 0.03	1.44 ± 0.03
Carr's index (%)	17.03 ± 0.01	22.92 ± 1.7	21.47 ± 0.02	0.30 ± 0.02
Angle of repose	36.5 ± 0.74	44.99 ± 1.52	41.67 ± 1.56	43.71 ± 0.47

## Data Availability

All the generated data used in this study could be available on request from the corresponding authors.

## Supplementary Materials

Supplementary Materials Figure S1: calibration curves of atomic absorption for various heavy metals.
