# Supplementary material for: Characterization of Boswellia rivae Engl Resin as a Potential Use for Pharmaceutical Excipient
Source: Biomed Res Int. 2022 Aug 8;2022:5791308. doi: 10.1155/2022/5791308 (PMC9377920; doi:10.1155/2022/5791308)
Supplement: Supplementary Materials — Figure S1: calibration curves of atomic absorption for various heavy metals. [file 5791308.f1.docx]

**Figure S1.** Calibration curves of atomic absorption for various heavy metals
